# Endothelial GC-A can be a therapeutic target for metabolic syndrome

**DOI:** 10.1186/2050-6511-14-S1-P73

**Published:** 2013-08-29

**Authors:** Takeshi Tokudome, Kentaro Otani, Ichiro Kishimoto, Yuanjie Mao, Kazuwa Nakao, Kenji Kangawa

**Affiliations:** 1 Department of Biochemistry, National Cerebral and Cardiovascular Center Research Institute, Osaka, Japan; 2 Department of Regenerative Medicine and Tissue Engineering, National Cerebral and Cardiovascular Center Research Institute, Osaka, Japan; 3 Department of Medicine and Clinical Science, Kyoto University Graduate School of Medicine, Kyoto, Japan; 4 National Cerebral and Cardiovascular Center Research Institute, Osaka, Japan

## Background

Atrial natriuretic peptide (ANP) has been used clinically for the treatment of heart failure patients in Japan, and also exhibits a variety of physiological effects through binding guanylyl cyclase-A (GC-A) receptor. We and other groups has been reported that GC-A is abundantly expressed in endothelial cells. In the present study, we explored the therapeutic potential of endothelial ANP/GC-A system for the treatment of metabolic syndrome.

## Methods

We generated endothelial cell-specific GC-A transgenic mice using Tie2 promoter and enhancer (EC-GC-A-Tg), inducible endothelial cell-specific GC-A transgenic mice (Inducible EC-GC-A-Tg), and also endothelial cell-specific GC-A knockout mice (EC-GC-A-KO). In addition, we used eNOS transgenic mice (eNOS-Tg). For the evaluation of blood pressure, telemetry system and tail-cuff method were used. Insulin resistance was evaluated by intra-peritoneal glucose tolerance test (IPGTT) and intra-peritoneal insulin tolerance test (IPITT).

## Results

The phenotypes of EC-GC-A-Tg were very unique. Systolic blood pressure in EC-GC-A-Tg was significantly lower compared with wild-type mice (WT). In addition, heat weight/body weight ratio and arterial elastance were smaller and lower in EC-GC-A-Tg compared with WT. Synchrotron radiation angiography showed decrease of basal vascular tone in EC-GC-A-Tg and increase in EC-GC-A-KO compared with in each control mice. Basal body weight of WT and EC-GC-A-Tg, flox mice and EC-GC-A-KO were comparable. However, after taking 8 weeks of high-fat diet, increase of body weight of EC-GC-A-Tg was significantly less than WT. On the other hand, EC-GC-A-KO showed more than increase of body weight. IPGTT and IPITT showed improvement of insulin tolerance in EC-GC-A-Tg, and worsen in EC-GC-A-KO compared with in each control mice. Interestingly, Inducible EC-GC-A-Tg showed less increase of body weight accompanied by aging. The body weight of eNOS-Tg was comparable with WT before and after taking 8 weeks of high-fat diet.

## Conclusion

These data suggest that endothelial ANP/GC-A system can be a therapeutic target for metabolic syndrome.

